# Specific markers and properties of synovial mesenchymal stem cells in the surface, stromal, and perivascular regions

**DOI:** 10.1186/s13287-018-0870-9

**Published:** 2018-05-02

**Authors:** Mitsuru Mizuno, Hisako Katano, Yo Mabuchi, Yusuke Ogata, Shizuko Ichinose, Shizuka Fujii, Koji Otabe, Keiichiro Komori, Nobutake Ozeki, Hideyuki Koga, Kunikazu Tsuji, Chihiro Akazawa, Takeshi Muneta, Ichiro Sekiya

**Affiliations:** 1 Center for Stem Cell and Regenerative Medicine, Tokyo Medical and Dental University, 1-5-45 Yushima, Bunkyo-ku, Tokyo, 113-8510 Japan; 2 Department of Biochemistry and Biophysics, Tokyo Medical and Dental University, Tokyo, Japan; 3 Department of Joint Surgery and Sports medicine, Tokyo Medical and Dental University, Tokyo, Japan; 4 Department of Cartilage Regeneration, Tokyo Medical and Dental University, Tokyo, Japan; 5 National Hospital Organization, Disaster Medical Center, Tokyo, Japan

## Abstract

**Background:**

Synovial mesenchymal stem cells (MSCs) are an attractive cell source for cartilage and meniscus regeneration. Synovial tissue can be histologically classified into three regions; surface, stromal and perivascular region, but the localization of synovial MSCs has not been fully investigated. We identified markers specific for each region, and compared properties of MSCs derived from each region in the synovium.

**Methods:**

The intensity of immunostaining with 19 antibodies was examined for surface, stromal, and perivascular regions of human synovium from six osteoarthritis patients. Specific markers were identified and synovial cells derived from each region were sorted. Proliferation, surface marker expression, chondrogenesis, calcification and adipogenesis potentials were compared in synovial MSCs derived from the three regions.

**Results:**

We selected CD55^+^ CD271^−^ for synovial cells in the surface region, CD55^−^ CD271^−^ in the stromal region, and CD55^−^ CD271^+^ in the perivascular region. The ratio of the sorted cells to non-hematopoietic lineage cells was 5% in the surface region, 70% in the stromal region and 15% in the perivascular region. Synovial cells in the perivascular fraction had the greatest proliferation potential. After expansion, surface marker expression profiles and adipogenesis potentials were similar but chondrogenic and calcification potentials were higher in synovial MSCs derived from the perivascular region than in those derived from the surface and stromal regions.

**Conclusions:**

We identified specific markers to isolate synovial cells from the surface, stromal, and perivascular regions of the synovium. Synovial MSCs in the perivascular region had the highest proliferative and chondrogenic potentials among the three regions.

## Background

Mesenchymal stem cells (MSCs) are an attractive cell source for cell therapies. These cells participate in tissue homoeostasis, remodeling, and repair by ensuring replacement of mature cells that are lost during the course of physiological turnover, senescence, injury, or disease [1]. Along with preclinical studies, a large number of clinical trials have been conducted for cardiovascular diseases, osteoarthritis, liver disorders, graft versus host disease (GvHD), respiratory disorders, spinal cord injury, and others [2]. MSCs are found not only in bone marrow but multiple adult tissues [3–5].

MSCs are defined as non-hematopoietic-lineage, plastic-adherent, self-renewing cells that can differentiate into chondrocytes, adipocytes and osteoblasts in vitro [6, 7]. Traditionally, the isolation of MSCs has relied on their adherence to plastic dishes and colony-forming ability in an unfractionated cell population. This technique may give rise to heterogeneous cell populations in MSCs. To better characterize this heterogeneity, surface markers have been investigated for bone marrow MSCs from the osteoblast region [8], endosteum region [9], and perivascular region [10].

Synovial MSCs have a higher chondrogenic potential than bone marrow MSCs [11]. Transplantation of synovial MSCs regenerated cartilage [12] and meniscus [13]. Synovial MSCs are clinically used for cartilage regeneration [14]. To prepare synovial MSCs, synovium is digested, and unfractionated synovial cells are expanded to form cell colonies of synovial MSCs [15, 16]. Synovial tissue can be histologically classified into three regions; surface, stromal, and perivascular regions [17]. If synovial cells can be obtained and synovial MSCs can be prepared from each region separately, more attractive synovial MSCs can be used in clinical therapies. This also provides important information on the physiological roles of cells in the synovium. The purpose of the present study was to identify specific markers for the isolation of synovial cells in the surface, stromal, and perivascular regions, and to compare properties of MSCs sorted by the specific markers.

## Methods

### Human synovium

This study was approved by the Medical Research Ethics Committee of Tokyo Medical and Dental University and all human study subjects provided informed consent. Human synovium was harvested from the knees of ten donors (59–85 years) with osteoarthritis during total knee arthroplasty.

### Transmission electron microscopy (TEM)

The specimens of synovial tissues were rapidly fixed in 2.5% glutaraldehyde in 0.1 M phosphate buffer for 2 h. The samples were washed with 0.1 M phosphate buffer, post-fixed in 1% OsO_4_ buffered with 0.1 M phosphate buffer for 2 h, dehydrated in a graded series of ethanol and embedded in Epon 812. Ultrathin sections at 90 nm were collected on copper grids, double-stained with uranyl acetate and lead citrate, and then examined by transmission electron microscopy (H-7100, Hitachi, Tokyo, Japan) [18].

### Immunostaining

Synovial tissues were rapidly embedded in OCT compound (Sakura Finetec Japan, Tokyo, Japan) and 4% carboxymethyl cellulose and were washed with 0.1% Tween-TBS. After blocking with Protein Block Serum-Free (Dako, Glostrup, Denmark), sections (5 μm thick) were incubated with 19 antibodies; CD90 (Becton, Dickinson and Company; BD, Franklin Lakes, NJ, USA), CD44 (BD), CD73 (BD), CD105 (BD), CD271 (Miltenyi Biotec, Bergisch Gladbach, Germany), CD140a (BD), CD140b (BD), CD29 (Merck Millipore, Darmstadt, Germany), CD49f (Merck Millipore), Ki67 (Dako), Proliferating Cell Nuclear Antigen (PCNA; Santa Cruz Biotechnology, Inc., Santa Cruz, CA, USA), CD55 (Miltenyi Biotec), CD31 (antibody derived from mouse (Dako) for IHC and sheep (R&D Systems, Minneapolis, MN, USA) for IF), CD146(BD), Laminin (Dako), Collagen type IV (Dako), Proteoglycan 4/Lubricin (PRG4; Santa Cruz Biotechnology), Hyaluronan synthase 1 (HAS-1; Santa Cruz Biotechnology) and HAS-2 (Santa Cruz Biotechnology), at 4 °C overnight. After washing three times, secondary antibodies (Chemmate Envision HRP-polymer, Dako) or anti-goat horseradish peroxidase (HRP)-conjugated secondary antibody (Dako) were added, followed by incubation for 30 min at room temparature. Staining was simultaneously developed in DAB+ solution (Dako), with counterstaining by hematoxylin. Samples were analyzed with a light microscope. The DAB positive intensity of surface and matrix protein expression in synovium was quantified with Image J software after immunostaining [19].

For fluorescence microscopy images, sections were first washed then incubated with Alexa Fluor 488- and/or 594-conjugated secondary antibodies (1:500; Thermo Fisher Scientific, Waltham, MA, USA) specific for the appropriate species for 1 h at room temperature. Samples were counterstained with 4,6-diamidino-2-phenylindole (DAPI; Wako, Osaka, Japan) and analyzed with a microscope (BZ-X700, Keyence Co., Ltd., Osaka, Japan).

### Flow cytometric isolation and analysis

Synovium was digested in a solution of 3 mg/mL collagenase (Sigma-Aldrich Japan, Tokyo, Japan) at 37 °C. After 3 h, the digested cells were filtered through a 70-μm cell strainer (Greiner Bio-One GmbH, Kremsmunster, Austria). The cells from six donors were harvested using a cell-dissociation buffer. Cells were suspended in HBSS at a density of 5 × 10^5^ cells/mL and stained for 30 min on ice with the antibodies. For cell isolation, cells were stained with CD31-PE-Cy7 (BD), CD45-PE-Cy7 (BD), CD235a-PE-Cy7 (BD), CD55-FITC (Miltenyi Biotec), CD90-PE (BD) and CD271-APC (Miltenyi Biotec) were used at day 0. Flow cytometric isolation of cell surface antigens were performed by a double-laser Aria 2 system (BD). For cell surface analysis, cells were stained with CD31-FITC (BD), CD45-FITC (BD), CD44-APC-H7 (BD), CD73-BV421 (BD), CD90-PE (BD), CD105-PerCP-Cy5.5 (BD), CD55-PE (BD), CD271-APC (Miltenyi Biotec), CD140b-PerCP-Cy5.5 (BD) and CD146-FITC (BD) at passage 3. Flow cytometric analysis of cell surface antigens was performed by a triple-laser FACS Verse system (BD). These data were analyzed using FlowJo software (Tree Star Inc., Ashland, OR, USA). Flow cytometric analyses were also performed for expanded cells at passage 3.

### Colony formation and proliferation ability of synovial MSCs

For proliferation assays, bulk and sorted synovial cells from four donors were plated on six wells at 2000 cells per 10 cm^2^ wells for 10 days in complete culture medium with 10% FBS (Thermo Fisher Scientific, Inc.) and 1% penicillin/streptomycin in alpha MEM (Thermo Fisher Scientific, Inc.). Cultured cells were harvested with 0.25% trypsin and 1 mM ethylenediaminetetraacetic acid (EDTA) (Thermo Fisher Scientific, Inc) at 37 °C for 5 min and counted with cell-counting plates. For colony formation assays, bulk and sorted synovial cells from four donors were plated as above. The dishes were stained with crystal violet at 14 days and the colony number was counted.

### Differentiation assay of synovial MSCs

For chondrogenic differentiation, cultured synovial MSCs from four donors at passage 2 were harvested using a cell-dissociation buffer as time 0 or cells at 48 h were harvested from preservation tubes after preservation. Then 2.5 × 10^5^ cells were transferred to a 15 mL tube (BD Falcon) and cultured in chondrogenic induction medium containing 10 ng/mL transforming growth factor-β3 (Miltenyi Biotec) and 1 μg/mL bone morphogenetic protein 2 (Medtronic, Minneapolis, MN, USA), in high glucose DMEM with 1% antibiotic-antimycotic (Thermo Fisher Scienific) which was changed every 3–4 days. After 21 days, chondrogenic differentiated cells were analyzed by safranin-o (Wako) staining.

For calcification induction, 100 cells were transferred to a 60 cm^2^ dish and cultured for 14 days in complete culture medium. Adherent cells were then cultured in osteogenic induction medium containing 50 μg/mL ascorbic acid 2-phosphate (Wako), 10 nM dexamethasone (Wako), and 10 mM β-glycerophosphate (Sigma-Aldrich), in complete culture medium, which was changed every 3–4 days. After 21 days, the differentiation of these cells into osteoblasts was assessed by alizarin red staining (Merck Millipore). To quantify the amount of alizarin red, the deposition was extracted by 10% (w/v) cetylpyridinium chloride (Sigma-Aldrich) in 10 mM sodium phosphate (pH 7.0) at room temperature for 1 h and the alizarin red stain in the extraction buffer was determined by measuring the optical density of the solution at 560 nm absorbance [15].

For adipogenic differentiation, adherent cells were cultured in adipogenic induction medium (Lonza, Basel, Switzerland), which was changed every 3–4 days. After 21 days, oil red-o staining (Muto Pure Chemicals, Tokyo, Japan) confirmed the differentiation of these cells into adipocytes. To quantify adipogenic ability, the amount of triglyceride was measured by adipogenesis assay kit (BioVision, Milpitas, CA, USA) in accordance with manufacturer’s instructions.

### RNA isolation and RT-PCR analysis

For chondrogenesis, six pellets from each donor were digested together. For calcification and adipogenesis, MSCs were plated at 50 cells/ cm^2^ in 145 cm2 plates and extracted from two dishes. Total RNA was extracted using RNeasy Mini Kit (Qiagen N.V., Venlo, Netherlands). Concentration and quality of the RNA were verified on a Quantus Fluorometer (Promega Co., Madison, WI, USA). The cDNA was synthesized with random hexamer primers from total RNA using the Transcriptor High Fidelity cDNA Synthesis kit (Roche Diagnostics, Basel, Switzerland). Real-time PCR was performed in a LightCycler 480 instrument (Roche Diagnostics). PCR reaction used the LightCycler 480 Probes Master. Relative amounts of mRNA were calculated and standardized as previously described [20]. The following TaqMan gene expression assay kits (Integrated DNA Technologies, IA, USA) were used as Hs.PT.39a.22214847 for *ACTB*, Hs.PT.58.38984663 for *SOX9*, Hs.PT.58.38672730 for *COL10A1*, Hs.PT.56a.742783 for *ACAN*, Hs.PT.56a.40555206 for *ALP*, Hs.PT.56a.19568141 for *RUNX2*, Hs.PT.58.25464465 for *PPARG*, Hs.PT.58.4022335.g for *CEBPA*, Hs.PT.58.3040231 for *GTF3A*, and Hs.PT.58.20087469 for *LPL*.

### Statistical analysis

All data were statistically evaluated with GraphPad Prism 6 (GraphPad Software, La Jolla, CA, USA). Data are expressed as mean ± SD. Each statistical analysis method is described in the legend. Two-tailed *P* values of < 0.05 were considered to be significant.

## Results

### TEM images for surface, stromal, and perivascular regions of synovium

**Fig. 1:**
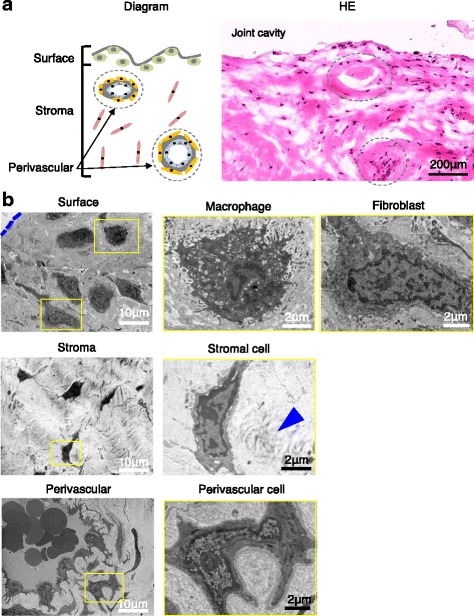
Surface, stromal, and perivascular regions of human synovium. **a** Diagram and H&E-stained histological image of synovium. **b** Transmission electron microscope (TEM) images. Surface of the synovium is indicated by *dashed line*. Around a stromal cell, collagen fibrils are indicated by an *arrowhead*

Synovium can be histologically classified into three regions; surface, stroma, and perivascular regions (Fig. 1a). These three regions had different ultra-microstructures. The surface region of the synovial membrane primarily consisted of macrophage and fibroblast cell components (Fig. 1b). The stromal region, defined as subsynovial tissue excluding the perivascular region, consisted of stromal cells with collagen fibrils. The perivascular region in synovial tissue contained blood vessels with perivascular cells.

### Immunological characterization for surface, stromal, and perivascular region of synovium

**Fig. 2:**
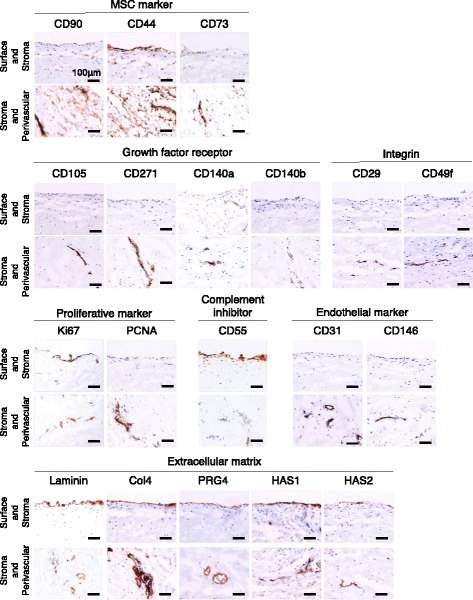
Immunostained images for surface, stromal, and perivascular regions of human synovium

To characterize the three regions immunologically, immunostaining with 19 antibodies was performed. We were able to detect the following proteins in the surface, stroma or perivascular region; MSC markers (CD90, CD44 and CD73), growth factor receptors of MSC markers (CD105, CD271, CD140a and CD140b), integrins (CD29 and CD49f), proliferative markers (Ki67, PCNA), complement inhibitor (CD55), endothelial markers (CD31 and CD146), and extracellular matrix (Laminin, Col4, PRG4, HAS-1 and HAS-2) (Fig. 2).

**Fig. 3:**
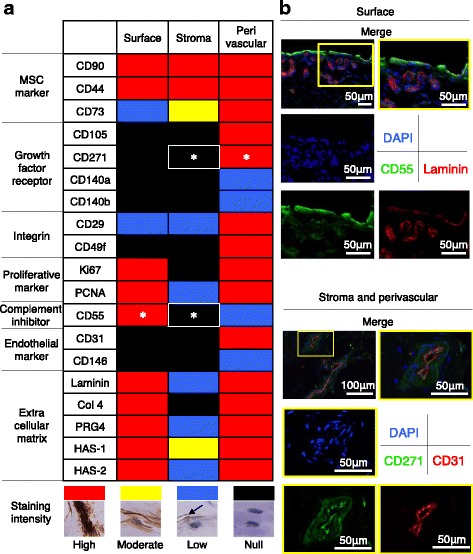
Immunological characterization for surface, stromal, and perivascular regions of human synovium. **a** Summary of immunological analysis for surface, stromal, and perivascular regions of human synovium. The staining intensity was subjectively graded into four colours; high (*red*), moderate (*yellow*), low (*blue*), and null (*black*) based on immunostained synovium derived from six patients. *; specific markers we selected for isolation of synovial cells derived from each region. **b** Fluorescent microscope images for synovium immunostained with specific markers for isolation of synovial cells derived from each region

The expression of proteins was region specific. Generally, in the surface region, MSC markers, proliferation markers, complement inhibitor, and extracellular matrices were highly expressed. In the stromal region, only MSC markers were highly expressed. In the perivascular region, most proteins we examined, with the exception of complement inhibitor, were highly expressed (Fig. 3a).

### Specific markers for isolation of synovial cells from surface, stromal, and perivascular regions

As specific markers for isolation of synovial cells from each region, we selected CD55 as a positive marker for synovial cells derived from the surface region, CD271 and CD55 as negative markers for synovial cells derived from the stromal region, and CD271 as a positive marker for synovial cells derived from the perivascular region (Fig. 3a). We confirmed the two markers by fluorescent immunostaining (Fig. 3b). CD55^+^ cells were found in the surface region and were colocalized with basement membrane positive for laminin. CD55 was also expressed on endothelial cells, but the level was lower than that of the surface region cells. CD271^+^ cells were confirmed in perivascular cells positive for CD31, while CD271 was not expressed in stromal cells negative for CD31.

### Ratio of synovial cells in the three regions by flowcytometric isolation

**Fig. 4:**
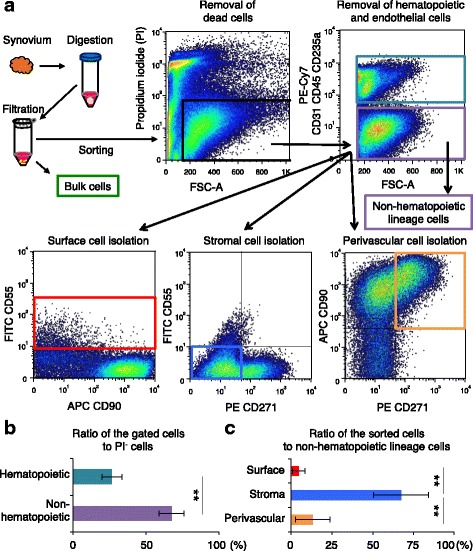
Ratio of the sorted surface, stromal and perivasucular cells. **a** Scheme for isolating surface, stromal and perivasucular cells. Synovium was digested and filtrated for bulk cells. From the bulk cells, dead cells were removed, then hematopoietic and endothelial cells were removed for non-hematopoietic lineage cells. From the non-hematopoietic lineage cells, surface cells were sorted for CD55, stromal cells were negative sorted for CD55 and CD271, and perivascular cells were sorted for CD90 and CD271. Non-hematopoietic lineage cells consisted of PI^−^ CD31^−^ CD45^−^ CD235a^−^ cells. **b** Ratio of the gated cells to PI^−^ cells. Bar shows mean ± SD. The analyzed synovium were obtained from eight donors were analyzed. **p < 0.01 by Wilcoxon matched-pairs signed rank test. **c** Ratio of the sorted cells to non-hematopoietic lineage cells. Bar shows mean ± SD. Synovia derived from eight donors were analyzed. **p < 0.01 by the Friedman test followed by Steel-Dwass multiple comparisons

From the non-hematopoietic lineage cells, synovial cells in the surface regions were sorted for CD55, those in the stromal region were negatively sorted for CD55 and CD271, and those in the perivascular region were sorted for CD271 (Fig. 4a). The ratio of the gated cells to PI^−^ cells was 25% in the hematopoietic cells and 70% in the non-hematopoietic cells (Fig. 4b). The ratio of the sorted cells to other non-hematopoietic lineage cells was approximately 5% in the surface region, 70% in the stromal region, and 15% in the perivascular region (Fig. 4c). The ratio of synovial cells derived from the stroma region was statistically higher than from the surface or perivascular regions.

### Proliferation and colony formation in the bulk and sorted synovial cells

**Fig. 5:**
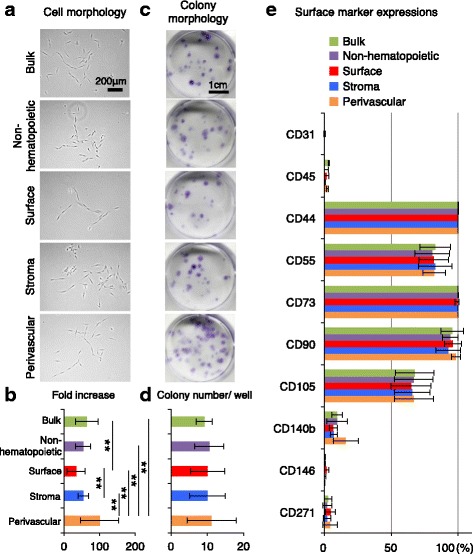
Proliferation and surface marker expression of colony-forming cells derived from non-sorted and sorted synovial cells. **a** Cell morphology of synovial cells 5 days after plating. **b** Fold increase of synovial cells. Bulk and sorted cells were plated at 200 cells/cm^2^ and cultured for 10 days. Bar shows mean ± SD. **p < 0.01 by the Friedman test followed by Steel-Dwass multiple comparisons (four donors, *n* = 6 for each donor). **c** Colony morphology of synovial cells. Bulk and sorted cells were plated on six-well plates at 100 cells/cm^2^, cultured for 14 days and stained with crystal violet. **d** Colony number/well. Bar shows mean ± SD (four donors n = 6 for each donor). **e** Surface markers of cultured MSCs. Bar shows mean ± SD (four donors)

We prepared five fractions of synovial cells; the bulk fraction, the non-hematopoietic and endothelial fraction, the surface fraction, the stromal fraction, and the perivascular fraction. No obvious morphological differences were observed among the five fractions (Fig. 5a). Proliferation was the highest in the perivascular fraction (Fig. 5b). Colony morphology appeared similar and no significant differences in colony number were obtained among the five fractions (Fig. 5c and d).

### Surface markers of colony-forming cells derived from the bulk and sorted synovial cells

Passage 3 colony-forming cells expressed CD44, CD73, and CD90 at high rates (over 90%); CD55 and CD105 at a moderate rate (50–90%); CD140b and CD271 at low rates (approximately 10%); and did not express CD31, CD45, and CD146 (less than 3%) (Fig. 5e). This expression pattern was similar to MSCs. Though synovial cells in the perivascular fraction were obtained after sorting with CD 271, the positive rate decreased to only 5% after colony formation.

### Differentiation of colony-forming cells derived from the bulk and sorted synovial cells

**Fig. 6:**
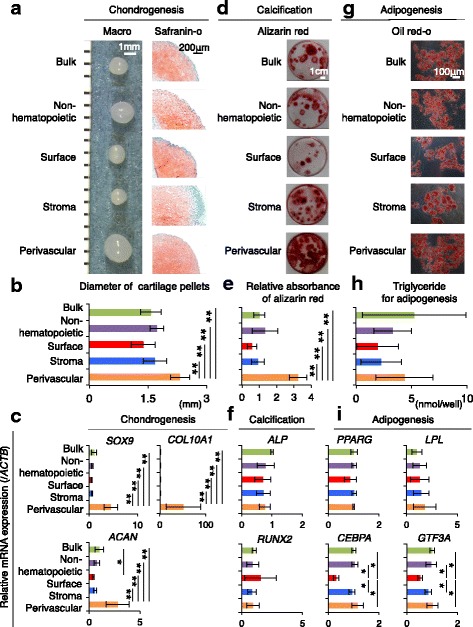
Differentiation ability of colony-forming cells derived from non-sorted and sorted synovial cells. **a** Chondrogenesis. Macroscopic images and histological sections stained with safranin-o are shown. **b** Diameter of cartilage pellets. Bar shows mean ± SD. **p < 0.01 by the Kruskal-Wallis test followed by Dunn multiple comparisons (four donors, *n* = 4 for each donor). **c** Relative mRNA expression per *SOX9*, Aggrecan *(ACAN)*, and *COL10A1* by real-time RT-PCR analyses using β-actin (*ACTB*) as housekeeping gene. Gene expression fold changes are shown and normalized to bulk as 1. Bar shows mean ± SD (three donors, n = 4 for each donor). **p < 0.01 by the Friedman test followed by Steel-Dwass multiple comparisons. **d** Calcification. Culture dishes stained with alizarin red are shown. **e** Quantification of alizarin red staining for calcification. Absorbance of alizarin red in the bulk group was set as 1. Bar shows mean ± SD. **p < 0.01 and *p < 0.05 by the Kruskal-Wallis test followed by Dunn multiple comparisons (four donors, n = 4 for each donor). **f** Relative mRNA expression per *ALP* and *RUNX2* by real-time RT-PCR analyses using β-actin (*ACTB*) as housekeeping gene. Gene expression fold changes are shown and normalized to bulk as 1. Bar shows mean ± SD (three donors, *n* = 2 for each donor). **g** Adipogenesis. Culture dishes stained with oil red-o are shown. **h** Quantification of triglyceride for adipogenesis. Bar shows mean ± SD (four donors, n = 4 for each donor). **i** Relative mRNA expression per *PPARG*, *LPL*, *GTF3A*, and *CEBPA* by real-time RT-PCR analyses using β-actin (*ACTB*) as housekeeping gene. Gene expression fold changes are shown and normalized to bulk as 1. Bar shows mean ± SD (three donors, n = 2 for each donor). *p < 0.05 by the Friedman test followed by Steel-Dwass multiple comparisons

After condrogenic induction, cell pellets from the five fractions were differentiated into cartilage that stained positive for safranin-o (Fig. 6a). The diameter of the cartilage pellets was the largest in the perivascular fraction among the five fractions (Fig. 6b). The perivascular fraction showed higher mRNA expression of *SOX9*, Aggrecan (*ACAN*), and *COL10A1* in the cartilage pellets (Fig. 6c).

After calcification induction, the colony-forming cells derived from synovial cells stained positive for alizarin red in the five fractions (Fig. 6c). Absorbance of alizarin red, used to quantify differentiation was also the highest in the perivascular fraction (Fig. 6d). Real-time RT-PCR analyses showed similar expression levels of *ALP* and *RUNX2* for the calcification observed for each region (Fig. 6f). No expression of osteogenic genes, such as *OPN*, *OCN*, *OSX*, and *DLX5,* was detected (data not shown).

After adipogenic induction, colony-forming cells derived from synovial cells stained positive for oil red-o in the five fractions (Fig. 6e). No significant differences were observed in triglyceride production among the five fractions (Fig. 6f). Real-time RT-PCR analyses showed similar expression levels of *PPARG* and *LPL* for the adipogenesis derived from each region (Fig. 6i). *GTF3A* and *CEBPA* expression was lower for the surface fraction than for the other four regions.

## Discussion

We used histological evaluations to classify the synovium into three regions: the surface, stroma, and perivascular regions. We then characterized the three regions immunologically by immunostaining with 19 antibodies. Based on the results, we selected CD55 as a positive marker for synovial cells derived from the surface region, CD271 and CD55 as negative markers for synovial cells derived from the stromal region, and CD271 as a positive marker for synovial cells derived from the perivascular region. The number of synovial cells derived from the perivascular region was much smaller than that derived from the stromal region, but the colony-forming cells derived from the perivascular region had higher proliferative and chondrogenic potentials.

We selected CD55, a complement inhibitor, as a specific marker for the surface region because activation of complement in the synovial membrane plays an important role in the pathogenesis of osteoarthritis [21]. CD55 is also recognized as a decay-accelerating factor (DAF) and is expressed in the synovial lining during inflammation. Synovial tissues derived from patients with osteoarthritis are exposed to inflammatory conditions in the knee joint, so the surface region is in constant contact with synovial fluid containing inflammatory cytokines. Analysis of MSCs positive for CD55 will therefore provide a better clarification of the pathology of osteoarthritis.

We selected CD271 as a specific marker for the perivascular region. CD271 is a low-affinity nerve growth factor receptor (LNGFR) and serves as one of the two receptor types for neurotrophins, a family of protein growth factors that stimulate the survival and differentiation of neuronal cells. These nerve cells in the intraarticular tissues are thought to be important in cartilage repair after cartilage damage, as demonstrated in mouse studies [22]. CD271 also serves as a marker of MSCs with high colony-forming ability in bone marrow and synovium [23–25]. The role of CD271 in the perivascular region of the synovium is unknown, but CD271 expression is possibly related to the pathophysiological response of vascular developments/neurogenesis associated with the synovitis of osteoarthritis. Functional analysis CD271 expression by the CD271^+^ cells in the synovial tissues will contribute to a greater understanding of the healthy and diseased state.

Though synovial cells in the perivascular fraction were obtained after sorting with CD 271, the positive rate decreased to only 5% after colony formation. This means that positive rate of CD271 decrease after expanding of the sorted cells. There are some reports describing that positive rate of CD271 decreased after expanding of CD271^+^ MSCs [23, 26]. Although the reason for the reduction of CD271 expression is not clear, one possibility is that the alteration of the environment from in vivo three-dimensional surroundings, in which CD 271-positive cells located at synovial perivascular region, to in vitro two-dimensional surroundings, in which CD 271-positive cells were expanded on culture dishes.

We selected CD55 and CD271 as negative specific markers for the stroma region, as our initial attempts to identify positive markers specific for the stroma region were unsuccessful. Other negative markers specific for the stroma region were CD55 (complement inhibitor), Ki67, and Col4 (extracellular matrix marker).

The surface marker expression pattern in the colony-forming cells derived from the bulk and sorted synovial cells in each group was similar to that observed for MSCs. These colony-forming cells also demonstrated multi-potentiality, indicating that indicate they were MSCs. This finding further confirmed that MSCs can be obtained from synovium, irrespective of sorting. Even without sorting, a small number of hematopoietic-lineage cells adhere to plastic dishes, and the rate of adhesion of hematopoietic-lineage cells further decreases after colony formation of the non-hematopoietic cells.

The MSCs derived from the perivascular fraction showed the largest cartilage pellets and the highest expression levels of *SOX9* and Aggrecan mRNA during chondrogenesis. The pellet size reflects the chondrogenic potential for each population of MSCs, whereas *SOX9* and Aggrecan mRNA expression reflects the chondrogenic potential of each individual MSC. Thus, the MSCs derived from the perivascular fraction had the highest chondrogenic potential, whether expressed per population or per single cell. However, these MSCs also showed the highest *COL10A1* mRNA expression, suggesting that their potential for hypertrophic chondrocyte differentiation though this was not evident in histological analyses.

The perivascular fraction also showed the highest absorbance of alizarin red, indicating pronounced calcification. This high absorbance might reflect the high number of cells present just before calcification induction, because MSCs derived from the perivascular fraction had a high proliferation capability. By contrast, the other five fractions showed equivalent mRNA expression of *ALP* and *RUNX2* and no mRNA expression of other osteogenic genes, such as *DLX5, OSX*, *OPN,* and *OCN* (data not shown). Based on these findings, we assume that this differentiation assay does not mimic the osteogenesis of MSCs, as we mentioned previously [20]. For this reason, we have used the term “calcification” rather than “osteogenesis” [27, 28].

The five fractions also showed equivalent triglyceride production and mRNA expression of *PPARG* and *LPL*, indicating similar adipogenesis activity. Conversely, mRNA expression levels of *GTF3A* and *CEBPA* were higher in the surface fraction than in the other fractions. *PPARG* and *CEBPA* are early markers and *LPL* and *GTF3A* are late markers of adipogenesis, so these contradictory expression patterns cannot be explained by different developmental stages. They may possibly reflect the complexity of molecular regulation of adipogenesis [29].

The perivascular fraction contained MSCs that were superior in proliferation and cartilage differentiation when compared to the MSCs in the other fractions. Caplan et al. recently advocated that MSCs are derived from pericytes, and our results support this hypothesis [30]. However, our results also demonstrated that MSCs are present in the surface and stromal regions, as well as in the perivascular region, although the MSCs from the surface and stromal regions showed poorer potentials for proliferation and cartilage differentiation.

The MSCs from the three synovial regions showed similar expression patterns of ten surface markers after expansion, but demonstrated different patterns of proliferation and differentiation. One reason for these differences might be that the three populations are still distinct, even though they show similar expression patterns for the ten surface markers. Other surface markers will therefore be useful to further distinguish these three populations. For example, Mabuchi et al. reported that the CD90^+^ and CD271^+^ fractions of bone marrow MSCs differed in their proliferation ability with and without expression of CD106 and CD49d [23]. Identification of surface markers or transcription factors to distinguish differentiation potentials among the three groups will be important for clarifying synovial MSC biology.

A higher proliferation and chondrogenic potential was observed in the present study MSCs derived from the perivascular region, although the number of sorted cells in the perivascular region was only approximately 20% of that in the stromal region. The MSCs derived from the stromal region showed poorer chondrogenic potentials; however, because greater numbers of MSCs could be prepared from that region than from the perivascular region, these differences in chondrogenic potential might be minimized. Therefore, from the standpoint of total cells harvested, the synovial stroma region could be a suitable MSC source.

The current study has three limitations that should be considered. First, the staining for the immunological characterization of surface, stromal, and perivascular regions of human synovium was graded subjectively and not objectively. Second, we selected CD55^+^ as the marker for synovial cells from the surface region, but because we investigated synovium derived from osteoarthritis patients, these cells may be representative of the inflammatory condition. Third, in vivo chondrogenesis was not evaluated.

## Conclusions

In conclusion, we selected CD55 as a positive marker for synovial cells derived from the surface region, CD271 and CD55 as negative markers for synovial cells derived from the stromal regions, and CD271 as a positive marker for synovial cells derived from the perivascular region. The number of synovial cells derived from the perivascular region was much lower than that derived from the stromal region, but the colony-forming cells derived from the perivascular region had higher proliferative and chondrogenic potentials.
